# Franglais a la mode moderne

**Published:** 1990-09

**Authors:** Jack Davies


**FRANGLAIS À LA MOôDE MODERNE**

A few years ago I was writing in this column about differences between languages. Dutch and Chinese are comparatively easy to learn. None the less, the various computer languages are another game altogether.

Recently a French colleague in pathology, who is also interested in the use of words and in the modern techniques of the assembly of data bases and word processing was kind enough to let me try out his own personal computer after it had been 'booted up' with CP/M and the French version of Messrs Ashton-Tate's d BASE II. (1–3.

Any beginner in computers can expect disasters in his prentice attempts at programming, or even in the basic steps in learning a new language. Usually they are just crude mistakes, and quite trivial.

Sometimes such mistakes have a quaint appeal. The last time this correspondent dared to speak in machine language was in Mallard BASIC (*Bristol Med-Chi J* 1986 **101** 62–63). Part of a later disastrous first screen assembly, admittedly in fractured d BASE II computerese Franglais, ran as follows:

. recall
*** AUCUNE BASE N'EST UTILUSEE, DONNEZ SON NOM:
. select
*** ERREUR DE SYNTAXE ***
?
*** DESIREZ-VOUS CORRIGER ET RECOMMENCER (Y/N)? REMPLACEZ
.n
*** CETTE COMMANDE NE PEUT ETRE UTILISEE ***
,....et enfin:
*** FIN DU TRAITEMENT. DORMEZ BIEN ***
.quit

There have occasionally been times in the distant past when both the French and English languages have seemed inadequate to express one's romantic feelings. Since logic is not all, were I French, the restricted if reasoned responses from their computers, would seem to have yet more syntactical and grammatical inadequacies. Their computers just ain't talking French. How do you express sentimental feelings when battered by a prompt:
'*** ERREUR DE SYNTAXE ***'?

However, nowadays it seems that English, French and even Franglais are mere past participles. Data bases, soft-ware and computers rule, and they are fast making their own brand-new languages. I only hope that our Editor of the nascent *West of England Medical Journal* will not pass over his own fierce red and blue pencils to a computer.

JACK DAVIES

**REFERENCES**

1. BYERS, R. A. (1982) Everyman's data base primer. Ashton-Tate, Culver, California.
2. DE PACE, M. (1984) Working with d Base II. Collins, London.
3. SIMPSON, A. (1985) Advanced techniques in d Base II. Sybex, Berkeley, California.

